# Microvascular, Biochemical, and Clinical Impact of Hyperbaric Oxygen Therapy in Recalcitrant Diabetic Foot Ulcers

**DOI:** 10.3390/cells14151196

**Published:** 2025-08-04

**Authors:** Daniela Martins-Mendes, Raquel Costa, Ilda Rodrigues, Óscar Camacho, Pedro Barata Coelho, Vítor Paixão-Dias, Carla Luís, Ana Cláudia Pereira, Rúben Fernandes, Jorge Lima, Raquel Soares

**Affiliations:** 1 RISE-Health, School of Medicine and Biomedical Sciences, Fernando Pessoa University, Fernando Pessoa Teaching and Culture Foundation, Avenida Fernando Pessoa 150, 4420-096 Gondomar, Portugal; 2 CECLIN—Centre of Clinical Studies, Hospital Fernando Pessoa, Avenida Fernando Pessoa 150 São Cosme, 4420-096 Gondomar, Portugal; 3 Fernando Pessoa-Biomedical Health Sciences (FPBHS), FP-I3ID—Instituto de Investigação, Inovação e Desenvolvimento, University Fernando Pessoa, Praça de 9 de Abril 349, 4249-004 Porto, Portugal; 4 Bomedicine Department, Faculty of Medicine, University of Porto, Alameda Prof Hernâni Monteiro, 4200-319 Porto, Portugalraqsoa@med.up.pt (R.S.); 5 Centro de Biotecnologia e Química Fina Laboratório Associado, Escola superior de Biotecnologia, Universidade Católica Portuguesa, R. de Diogo Botelho 1327, 4169-005 Porto, Portugal; 6 i3S—Instituto de Investigação e Inovação em Saúde, University of Porto, 4200-135 Porto, Portugal; 7 Hyperbaric Medical Unit, Unidade Local de Saúde de Matosinhos, E. P. E (Entidade Pública Empresarial), R. de Dr. Eduardo Torres, 4464-513 Senhora da Hora, Portugal; 8 Instituto de Ciências Biomédicas Abel Salazer, R. de Jorge de Viterbo Ferreira 228, 4050-313 Porto, Portugal; 9 Hospital de Santo António, Unidade Local de Saúde de Santo António, Largo Prof. Abel Salazar, 4099-001 Porto, Portugal; 10 Internal Medicine Department—Centro Hospitalar de Vila Nova de Gaia/Espinho EPE, 4434-502 Vila Nova de Gaia, Portugal; 11 IPATIMUP—Institute of Molecular Pathology and Immunology of the University of Porto, 4200-465 Porto, Portugal; 12 Pathology Department, Faculty of Medicine, University of Porto, Alameda Prof Hernâni Monteiro, 4200-319 Porto, Portugal

**Keywords:** diabetes, diabetic foot, foot ulcer, hyperbaric oxygen therapy, angiogenesis, microvessel density

## Abstract

Background: Diabetic foot ulcers (DFUs) are a serious complication of diabetes and are often difficult to treat. Hyperbaric oxygen therapy (HBOT) has been proposed as an adjunctive treatment to promote healing, but its long-term clinical and biological effects remain insufficiently characterized. This study aimed to evaluate the impact of HBOT on systemic biomarkers, local microvasculature, and clinical outcomes in patients with DFUs. Methods: In this non-randomized prospective study, 20 patients with ischemic DFUs were followed over a 36-month period. Fourteen received HBOT in addition to standard care, while six received standard care alone. Clinical outcomes—including DFU resolution, recurrence, lower extremity amputation (LEA), and mortality—were assessed alongside systemic inflammatory and angiogenic biomarkers and wound characteristics at baseline and at 3, 6, 12, and 36 months. CD31 immunostaining was performed on available tissue samples. Results: The two groups were comparable at baseline (mean age 62 ± 12 years; diabetes duration 18 ± 9 years). At 3 months, the HBOT group showed significant reductions in erythrocyte sedimentation rate and DFU size (*p* < 0.05), with downward trends observed in C-reactive protein (CRP), vascular endothelial growth factor (VEGF), and placental growth factor (PlGF), and an increase in stromal-derived factor-1 alpha (SDF1-α). No significant changes were observed in the control group. CD31+ microvessel density appeared to increase in HBOT-treated DFU tissue after one month, although the sample size was limited. Patients receiving HBOT had lower rates of LEA and mortality, improved wound healing, and sustained outcomes over three years. DFU recurrence rates were similar between groups. Conclusions: HBOT was associated with improved wound healing and favorable biomarker profiles in patients with treatment-resistant ischemic DFUs. While these findings are encouraging, the small sample size and non-randomized design limit their generalizability, highlighting the need for larger, controlled studies.

## 1. Introduction

Diabetes mellitus (DM) is one of the most prevalent metabolic disorders worldwide, currently affecting an estimated 382 million individuals [1,2]. Its global burden is largely attributed to its many complications, including diabetic foot ulcers (DFUs), which develop in approximately 10–25% of patients. Among these cases, 50–70% will experience recurrence within five years, and impaired healing may result in lower extremity amputation (LEA) in 7–20% of patients [3,4,5,6].

DM contributes to defective wound healing due to alterations in both the micro- and macrovasculature, as well as disruptions in the cellular and molecular environment, where neuropathy and infection play significant roles [4,7].

Wound healing occurs in four distinct but overlapping phases, including hemostasis, inflammation, proliferation, and tissue remodeling [8,9], all of which are highly dependent on adequate tissue oxygenation [8,10,11,12]. As such, sufficient tissue vascularization to ensure oxygen availability is critical for the entire healing process.

In adults, neovascularization occurs through two main mechanisms: angiogenesis, via the migration and differentiation of resident endothelial cells, and vasculogenesis, involving bone marrow-derived undifferentiated angioblasts or endothelial progenitor cells (EPCs) [13,14].

Interestingly, angiogenesis is upregulated in some diabetic organs (e.g., the retina), while it is impaired in others, such as the distal microvasculature of the lower limbs, in a phenomenon referred to as the “diabetic paradox” [7,15].

DFUs frequently progress to chronic wounds, defined as lesions that fail to heal within 4–6 weeks despite standard therapy. Local hypoxia is a hallmark of chronic wounds, with tissue oxygen pressure (pO_2_) often ranging between just 10 and 30 mmHg, far below the optimal 50–100 mmHg required for proper healing [10,15]. These hypoxic conditions have prompted the clinical use of hyperbaric oxygen therapy (HBOT), which involves administering 100% oxygen in a pressurized chamber, typically at 1.5 to 3.0 ATA (152–304 kPa). Previous studies have shown that HBOT can significantly reduce the risk of major LEA in patients with chronic DFUs [16].

Animal studies further support these findings, suggesting that HBOT enhances healing by stimulating neovascularization through various mechanisms—including increased synthesis of growth factors within the wound bed and recruitment of EPCs [4,7,17,18]. Still, clinical data in human patients with diabetes and active DFUs remain limited.

Despite growing interest in HBOT as an adjunct therapy for DFUs, the current evidence remains inconclusive due to several methodological limitations in earlier studies. Most existing randomized controlled trials (RCTs) and controlled clinical trials (CCTs) have involved small sample sizes and short follow-up durations, often failing to assess long-term outcomes such as ulcer recurrence or delayed amputations. Moreover, many studies lacked blinded outcome assessment or standardized wound measurement protocols, introducing bias and variability.

In light of these gaps, this study aimed to evaluate the clinical and biological effects of HBOT as an adjunctive therapy in patients with active DFUs. Specifically, we compared patients receiving standard care alone to those receiving HBOT in addition to standard care, assessing (a) biomarkers (including systemic biochemical, angiogenic, and vasculogenic markers) as well as DFU area, depth, and volume at 3 months; (b) clinical outcomes (complete healing, LEA, and mortality) at 3, 6, and 12 months; and (c) long-term outcomes (DFU recurrence, LEA, and mortality) at 3 years.

This study aims to address prior limitations by providing long-term (36 month) clinical, biochemical, and histological data using blinded outcome evaluations and objective digital wound measurement tools—offering a more comprehensive and reliable real-world assessment of HBOT’s effectiveness in DFU management.

## 2. Materials and Methods

### 2.1. Study Design and Participants Selection

A non-randomized, prospective observational study was conducted in a real-world clinical setting at a tertiary care diabetic foot outpatient clinic, staffed by a multidisciplinary team. Due to ethical and logistical constraints, randomization was not feasible. Patients were allocated based on clinical criteria, contraindications to HBOT, or informed refusal. Although the study was not randomized, baseline characteristics between the HBOT and non-HBOT (comparison) groups were statistically comparable (see Table 1). No *a priori* sample size calculation was performed, as this was an exploratory study based on consecutive patient enrollment.

The study followed the principles outlined in the Declaration of Helsinki and received approval from the institution’s Ethics Committee. Written informed consent was obtained from all participants. No financial compensation was provided.

All patients referred to the center with chronic diabetic foot ulcers (DFUs) were screened for eligibility. Individuals aged ≥ 18 years with an active DFU, defined as a full-thickness skin defect distal to the malleoli that persisted for more than 15 days [19], were considered. Patients whose ulcers showed no significant improvement (defined as no healing or <30% reduction in area) after eight weeks of optimized standard treatment (including angioplasty and/or revascularization, if needed) were consecutively proposed for HBOT.

Participants were enrolled over a 15-month period and followed for up to 3 years or until death.

The decision to offer HBOT was made collaboratively by the multidisciplinary team and approved by the hospital’s financial department. Patients selected for HBOT were those deemed most likely to benefit from the therapy. Exclusion criteria included patients who were bedridden, fully dependent in daily living activities, or declined participation. HBOT was considered a last-resort therapy in patients who had no improvement despite maximized macrovascular blood flow. Based on treatment status, participants were categorized into two groups: (1) HBOT group: patients receiving adjunctive hyperbaric oxygen therapy; (2) Non-HBOT group: patients who either refused HBOT or had contraindications, receiving standard care only. HBOT was administered at a designated regional center following a standardized protocol: 80-min sessions at 2.4 ATA (243.2 kPa), once daily, five days per week, up to a maximum of 100 sessions.

Throughout the study period, all participants received standard wound care delivered by a team independent of the research investigators. Standard care included regular debridement, moist dressings, infection control, and glycemic optimization, per institutional protocols. This care was applied consistently across both groups, with HBOT added as an adjunct in the intervention group. For patients with multiple ulcers, only the largest DFU was tracked using wound area, depth, and volume measurements. After completing the HBOT protocol, patients continued follow-up in standard outpatient care, including routine wound assessments and interventions as clinically necessary.

Laboratory biomarkers were assessed at baseline and again at 3 months. DFU healing progress (area, depth, volume) and the occurrence of LEA or death were recorded at 3, 6, and 12 months. Long-term follow-up continued up to 3 years or until death, with documentation of DFU recurrence or LEA (see Table 1).

### 2.2. Participants and Diabetic Foot Ulcer Characteristics

At enrolment, the following demographic characteristics were collected: age at the time of inclusion; gender; DM type (classified according to the World Health Organization definition [20]), duration (in years), and treatment (oral anti-diabetic agents or insulin); metabolic control [through glycated hemoglobin (HbA1c)]; smoking habits; presence of any DM related complications [namely, retinopathy, nephropathy, neuropathy, cerebrovascular, cardiovascular, and/or peripheral arterial disease (PAD) and metabolic], were registered in accordance to the definition used in the Diabetes Complications Severity Index created by Young et al. [21].

For foot characterization, we used the presence of peripheral arterial disease (PAD), only when one or fewer pedal pulses were palpable on the DFU foot [22] and/or the ankle-brachial index (ABI) was inferior to 0.8 [23]; transcutaneous partial pressure of oxygen (TcPO_2_), determined by measuring once at two points peri-DFU and reporting the highest value; diabetic peripheral neuropathy (DPN), defined as inability to feel the Semmes–Weinstein monofilament (SWM) at one or more of four specific sites on the foot [24]; and previous DFU or LEA. We also recorded the ulcer area (in cm^2^), reported duration (in months), location, Texas University Classification (TUC) [25], number of DFUs, and the presence or absence of infection.

DFU photographic and dimensional records (area, maximum and mean depths, and volume) were recorded using the Silhouette Mobile™ (Lindon, UT, USA) digital wound assessment system (Aranz Medical, Christchurch, New Zealand), which captures photographic documentation.

It was considered complete healing when the DFU presented full epithelialization without the need of further dressing [26]. Minor LEA was defined as amputation distal to or including the forefoot, and major LEA was considered amputation above or by the ankle [27]. Recurrence was considered a new DFU irrespective of location and time since previous DFU.

### 2.3. Laboratory and Molecular Analysis

In both groups (HBOT and non-HBOT), blood samples were collected at baseline and at 3 months. In addition, tissue samples from DFU debridement were collected at baseline (0 months) and after 1 month. Blood was drawn into both EDTA (ethylenediamine tetraacetic acid) tubes and serum separator tubes containing gel and clot activator.

A full blood count was performed using an automated hematology analyzer XE 2100 or 5000 (Sysmex Corporation, Norderstedt, Germany).

Serum was analyzed for glucose, urea, creatinine, total proteins, albumin, lipid profile, uric acid, and C-reactive protein (CRP) with the Cobas 8000c701 (Roche Diagnostics; Hitachi High-Technologies Corporation, Tokyo, Japan).

HbA1c was determined using high-pressure chromatography using the Horiba Medical G7 device (Horiba Medical; Horiba ABX SAS version 22_01, Kyoto, Japan).

Microalbuminuria level was evaluated using immunoturbidimetry with Cobas 6000 c501 analyzer (Roche Diagnostics; Hitachi High-Technologies Corporation, Tokyo, Japan).

Angiogenic [vascular endothelial growth factor (VEGF), placental growth factor (PlGF)] and vasculogenic [stromal-derived factor-alpha (SDF1-α)] markers were determined by enzyme-linked immunosorbent assay (ELISA) multiplex using Quantikine ELISA Immunoassay kits [R&D Systems, Abingdon, UK], according to the manufacturer’s instructions.

### 2.4. Immunohistochemistry Assays

Ulcer bed tissue was obtained during DFU debridement procedures for histological and immunohistochemical analyses. Specimens were fixed in 10% neutral-buffered formalin and subsequently embedded in paraffin. Serial sections (3 μm thick) were prepared and either stained with hematoxylin and eosin (H&E) or processed for immunohistochemical detection of capillary endothelial cells.

For immunostaining, endogenous peroxidase activity was quenched using 4% hydrogen peroxide in phosphate-buffered saline (PBS) for 30 min at room temperature. Antigen retrieval was performed by heating sections in 10 mM citrate buffer (pH 6.0) at 98 °C. Non-specific binding was blocked by incubating the sections with 10% bovine serum albumin (BSA) in PBS for 1 h. Sections were then incubated overnight at 4 °C with a primary antibody against cluster of differentiation 31 (CD31; 1:100 dilution; Abcam, Cambridge, UK). Then, anti-rabbit secondary antibody (1:200) (Santa Cruz Biotechnology, Dallas, TX, USA) was applied for 30 min. The avidin biotin complex (ABC) method (Vectastain ABC kit, Vector, Burlingame, CA, USA) was used according to the manufacturer’s instructions. The antigen–antibody reaction was developed using diaminobenzidine (DAB) (DAB substrate kit, Abcam, Cambridge, UK) as a peroxidase substrate, rendering CD31-positive cells with a brown staining. Sections were counterstained with hematoxylin (Sigma-Aldrich, Algés, Portugal), dehydrated, and coverslipped. CD31-expressing microvessels were counted in the three most vascularized areas with magnification of 200 x, and the data were averaged and normalized to the total area of the tissue section. Any positive-staining endothelial cell or endothelial cell cluster that was separated from adjacent microvessels was considered an individual vessel [28].

Every laboratory and molecular studies were performed by investigators blinded to the subjects’ group allocation.

### 2.5. Statistical Analysis

Continuous variables were described as mean ± standard deviation (SD) for normally distributed data or as median and range for non-normally distributed data. Normality was assessed through analysis of skewness and kurtosis, as well as visual inspection of histograms.

Between-group comparisons were performed using the independent samples *t*-test for normally distributed variables or the Mann–Whitney U test for non-parametric data. Within-group comparisons between two time points were conducted using the paired *t*-test or the Wilcoxon signed rank test, as appropriate.

Categorical variables were summarized using frequencies and percentages. Associations between categorical variables were assessed using the Chi-square (Χ^2^) test or Fisher’s exact test, when applicable.

All statistical tests were two-sided, and *p*-values < 0.05 were considered statistically significant. Missing data, primarily due to amputation or death, were handled using complete case analysis at each time point. No data imputation or sensitivity analysis was conducted. Statistical analysis was performed using the IBM SPSS version 22.0 (Chicago, IL, USA).

## 3. Results

### 3.1. Participants’ Characteristics

A total of 20 patients were included in the study: six in the non-HBOT DFU and 14 in the HBOT DFU groups described in Table 2. The DFU participants were allocated not to undergo HBOT due to refusal of treatment in five patients and one for presenting a contra-indication.

Taken the whole sample of patients included into account, the mean age was of 62 ± 12 years with a DM duration 18 ± 9 years, and the majority were male (85%), with type 2 DM (95%) treated with insulin (70%), with visual and physical impairment. Concerning DM complications, most had retinopathy (90%), PAD complications (85%) and neuropathy (85%). The mean complication count was four.

All ulcers included in the study were of ischemic or mixed etiology. Almost all DFUs reached the bone and were infected and ischemic as reflected by the Texas University Classification grade III D. Several were post-minor LEA and 25% were located in the toes.

There were no significant differences in any baseline characteristic when comparing both groups. The HBOT group tended to have more frequently altered SWM sensation but less frequently previous DFU history and digital DFU (*p* ≤ 0.1).

The HBOT group had a mean number of 86 sessions, and all patients completed the full prescribed treatment protocol without early discontinuation.

### 3.2. DFU Evolution and Clinical Outcome in the Short Term

At baseline, DFUs had equivalent dimensions in the two groups of participants (Table 3). Only one subject remained alive and without major LEA at the 3rd month in the non-HBOT DFU. Thus, no comparison was possible between baseline and this time point. This substantial reduction in sample size in the comparison group limits the robustness and generalizability of longitudinal outcome comparisons. Nevertheless, this patient exhibited a larger and deeper DFU when comparing to the median values in the HBOT DFU group.

All DFU measurements, including mean depth, maximum depth, and volume, showed or tended to show significantly lower values at 3 months in the HBOT group compared to baseline (Table 2).

At the 3-month follow-up, significant differences in clinical outcomes were observed between patients who received HBOT and those in the non-HBOT group (Table 4). In the non-HBOT group, four patients (67%) underwent lower extremity amputation (LEA), including three major amputations and one midfoot amputation. One patient died in the early postoperative period due to surgical complications, and another died from lung cancer. Additionally, one subject died after refusing major LEA. In total, 50% of patients in the non-HBOT group died during the study period.

In contrast, all participants in the HBOT group showed improvement or achieved complete healing at 3 and 6 months, and none required lower extremity amputation. However, three patients (21%) died within 12 months due to causes unrelated to DFU. Statistically significant differences in outcomes were observed between the HBOT and non-HBOT groups at all assessed time points. Moreover, deaths in the non-HBOT group occurred earlier in the study period (by month 3), whereas in the HBOT group, they occurred later (by month 12).

### 3.3. Metabolic Parameters

At the beginning of the study, the whole sample presented a mean HbA1c of 8 ± 1.8%, total cholesterol of 168 ± 52 mg/dL and triglycerides of 131 ± 56 mg/dL (Table 5). These findings indicated that glycemic control and lipid profile were reasonably controlled in both groups.

At baseline, there were no significant differences between HBOT and non-HBOT groups when comparing any of the metabolic markers. In the non-HBOT group, after 3 months, there was a significant reduction in the erythrocyte sedimentation rate (ESR) and an increase in uric acid.

In the group undergoing HBOT, after 3 months of therapy, statistically significant lower values of low-density lipoprotein (LDL), platelets, and ESR were observed. In addition, total cholesterol and CRP tended to decrease (*p* ≤ 0.1).

At 3 months, the HBOT group had a lower CRP (*p* < 0.05) value and tended to present lower HbA1c and leukocytes values (*p* < 0.1) when compared to the non-HBOT group.

No statistically significant differences were found for the remaining biochemical parameters (values are described in Table 5).

### 3.4. Microvascular Markers and Vessel Density

At baseline, vasculogenic biomarker SDF1-α tended to present a lower value in the HBOT group (*p* < 0.1). On the other hand, angiogenic biomarkers VEGF and PlGF values were similar between groups.

There was no modification, with statistical significance, in the non-HBOT group regarding both angiogenic or vasculogenic biomarkers after 3 months of follow-up.

In the HBOT group, after 3 months of therapy, the values of VEGF and PlGF tended to decrease, whereas the vasculogenic biomarker SDF1-α tended to increase (*p* ≤ 0.1).

Comparing the two groups at 3 months, there was a trend to lower levels of VEGF and higher levels of SDF1-α in the HBOT group, not reaching statistical significance (*p* ≤ 0.1).

Since DFU bed tissue was collected only during debridement, not all samples contained viable tissue. As a result, immunohistochemistry for CD31-expressing microvessels, with samples paired by subject, was only feasible in three patients from the non-HBOT DFU group and six patients from the HBOT DFU group.

**Figure 1:**
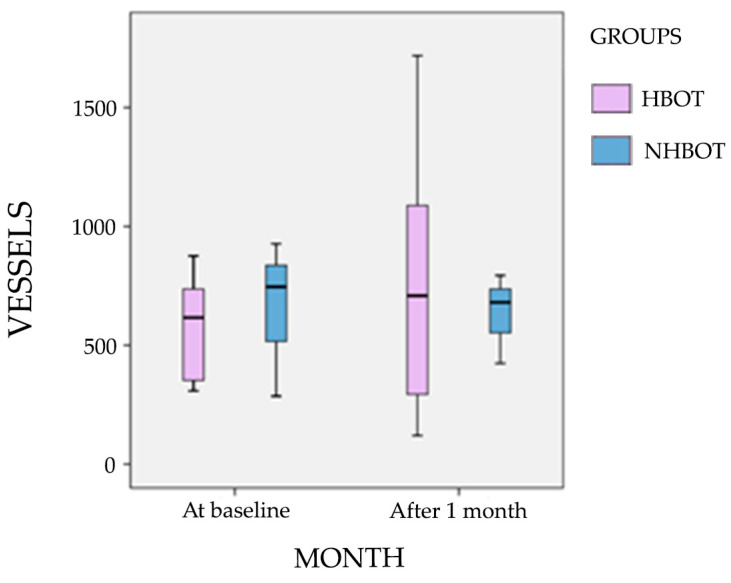
Boxplot of number of vessels at baseline and after 1 month in patients with (HBOT) and without hyperbaric oxygen therapy (NHBOT).

**Figure 2:**
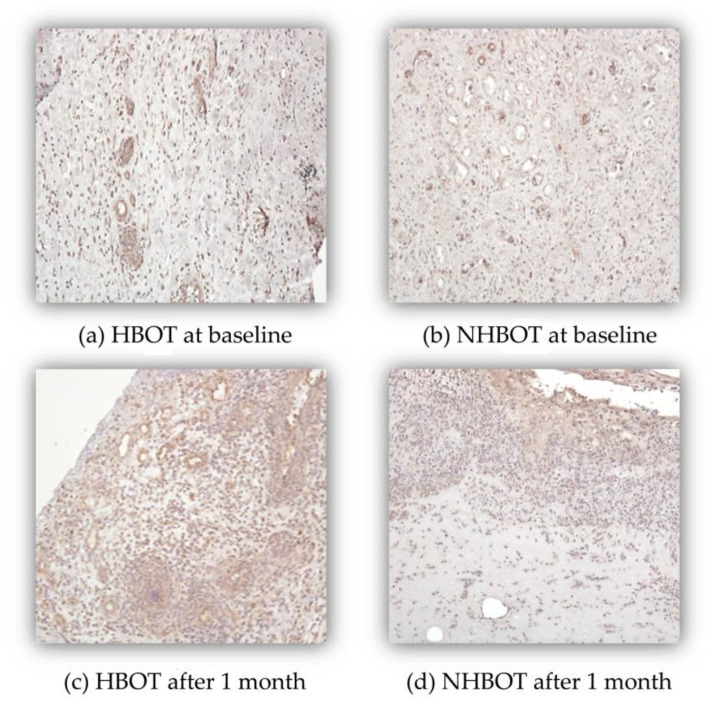
Microvessel density as evaluated using CD31 immunostaining at baseline and month 1 in patients with active DFU. (**a**) Patient treated with HBOT at baseline; (**b**) Non-HBOT patient at baseline; (**c**) Patient treated with HBOT at one month after treatment initiation; (**d**) Non-HBOT patient one month after treatment initiation. Magnification 200×.

Histological analysis showed a trend for increased CD31+ microvessel density in the HBOT DFU after 1 month of treatment (617 vs. 709 microvessels, as counted across three fields under 200× magnification). In contrast, a slight reduction was observed in the number of microvessels for the non-HBOT DFU patients (746 vs. 680 microvessels, as counted across three fields under 200× magnification), not achieving, however, statistical significance (Figure 1). Figure 2 illustrates the immunohistochemistry results. These observations are based on a small number of available samples, which limits the statistical power of this comparison.

### 3.5. Long-Term Clinical Outcome

From baseline to 3 years of follow-up, five patients (25%) of the sample experienced DFU recurrence, three underwent major LEA, one had a midfoot LEA (20% in total), and seven patients (35%) died.

Among the three subjects in the non-HBOT group who survived the first year of follow-up, none experienced DFU recurrence, underwent further major LEA, or died by the end of the three-year follow-up.

In the HBOT group, 11 subjects remained alive at the one-year mark. By the end of the three-year follow-up, two additional deaths had occurred (18%). Five subjects (46%) developed a new DFU, but none required major LEA.

No statistically significant differences between groups were observed for any of these outcomes when analyzing the remaining sample.

However, when using the initial sample, the significant difference in LEA rates found at 12 months persisted over the three-year period (HBOT 0% vs. non-HBOT 67%, *p* = 0.003). In terms of DFU recurrence (HBOT 36% vs. non-HBOT 0%, *p* = 0.3), no statistically significant difference was found. In contrast, mortality rates differed significantly (HBOT 29% vs. non-HBOT 50%, *p* = 0.003).

## 4. Discussion

Several studies have addressed HBOT impact on DFU healing [16]. However, this is the first study to simultaneously evaluate and compare microvascular and biochemical markers as well as long-term clinical outcome in subjects with DFU undergoing or not undergoing HBOT.

After 12 months of HBOT, all subjects included in the present study with recalcitrant DFUs showed improvement or complete DFU healing, and none underwent LEA. However, three patients (21%) died. Comparatively, in the non-HBOT group, at 12 months, four underwent LEA (67%), and three (50%) died. These results presented statistical significance.

When extending the follow-up to three years, five patients in the HBOT group (36%) developed a new DFU, none required a LEA, and four died (29%). In the non-HBOT group, no new DFUs were reported, four subjects (67%) required LEA, and three died (50%). Statistical significance was maintained only for the LEA outcome.

It is important to highlight that, in the non-HBOT group, deaths occurred earlier (at 3 months compared to 12 months or later in the HBOT group). Furthermore, after 12 months, two of the surviving non-HBOT subjects had severely limited physical activity, as both had undergone major LEA.

Our sample population presented mean values for age, gender distribution, DM duration, and HbA1c that were comparable to those reported in RCTs [29,30,31,32,33,34,35,36,37] and non-randomized trials (NRTs) [38,39]. The median TcPO2 was lower than in most studies [34,40,41], and the DFUs were deeper than those reported in RCTs [25,31,34], but similar to those in NRTs [39,42]. Therefore, our participants present a baseline prognosis comparable to or worse than those of subjects included in studies of similar evidence level.

When compared with the literature, the clinical outcome rates observed in the present study were equivalent (within the 95% CI) to those reported in both RCTs and NRTs with follow-up periods of at least 12 months, for both the HBOT and non-HBOT groups [29,30,31,35,38,40,42].

Our findings showed that patients who underwent HBOT presented lower ESR and CRP levels. These findings support the exiting evidence that HBOT contributes to a reduction in infection and systemic inflammatory response [43,44].

Ischemic and mixed DFU characteristically present hypoxic conditions and chronic inflammation. Another HBOT effect widely reported in the literature is the improvement of impaired vascularization within DFU tissues. Accordingly, we evaluated whether HBOT influenced systemic levels of vascular endothelial growth factor (VEGF) and placental growth factor (PlGF). After three months of HBOT, both VEGF and PlGF levels showed a decreasing trend, which may reflect a reduction in tissue hypoxia and a concurrent decline in inflammatory mediators.

SDF1-α is involved in the recruitment of circulating bone marrow-derived EPCs, which are known to be reduced in diabetes. We further investigated this growth factor in the serum of the individuals with DFU, who were either exposed or not to HBOT. In the HBOT group, SDF1-α levels increased by month 3. We hypothesized that HBOT promotes an increase in SDF1-α, potentially leading to a rise in the number of circulating EPCs [45].

Conversely, no changes in these angiogenic and vasculogenic markers occurred in the non-HBOT group.

DFU healing is closely associated with tissue vascularization. To further explore the effect of HBOT at the tissue level, we evaluated the number of vessels within the DFU tissue. We observed that, despite a reduction in systemic VEGF and PlGF levels, DFUs exhibited a trend toward an increased median microvessels count one month after treatment initiation in the HBOT group, while the opposite trend was noted in the non-HBOT DFUs. These findings confirm that HBOT enhances local vascularization within the DFU tissue.

These contradictory results, reduced VEGF serum levels alongside increased microvessel density in DFU of HBOT-treated subjects, can be explained by the fact that local tissue environment plays a pivotal role in the angiogenic process in individuals with diabetes [46]. This interpretation aligns with the well-established angiogenic paradox hypothesis, which highlights that the same patient may present exacerbated angiogenesis in organs like retina and kidney, and simultaneously angiogenesis impairment in others (e.g., limbs and myocardial ischemia) [47,48]. In addition, since VEGF is upregulated by hypoxia and by the inflammatory environment, the observed VEGF reduction may reflect a decrease in hypoxia and in the inflammatory environment, hallmarks of the diabetic foot milieu, coinciding with the improvements in the local microvasculature structure [49].

For ethical reasons, it was not possible to perform full-thickness DFU biopsies. Instead, small tissue fragments were collected during wound debridement procedures. Therefore, not all collected samples were fully representative of DFU wound bed, limiting the accuracy of microvessel density.

Infection and PAD, along with wound depth, are considered by several classifications as the most important prognostic factors in DFU assessment [50]. HBOT improves circulation by promoting microvascularization and optimizing oxygen delivery, thereby ameliorating leucocytes’ function and augmenting anti-bacterial response. We believe that such mechanisms are responsible for the good clinical outcomes observed in our study and are reflected in the results.

At the time of recruitment, DFUs were comparable between groups. In the HBOT group, all DFU-related measures showed a reduction at 3 months. In addition, at every assessed time point, the HBOT group achieved better outcomes when compared to those with not receiving such adjunctive therapy. Significantly lower rates of major LEA and death were observed, alongside longer survival rates. Compared to the available literature [29,31,38,39,40,42], it is noteworthy that our patients underwent to a higher number of sessions (mean of 86), which may have positively affected the results.

Remarkably, only one subject experienced a minor side effect due to HBOT (ear barotrauma), which resolved with appropriate treatment.

In DFUs, it is important to understand the metabolic environment for addressing factors that promote or impede healing. The combination of chronic low oxygen levels, ongoing inflammation, and a damaged wound environment in DFUs can lead to a shift in the tissue metabolism [51]. Due to poor blood flow, the oxygen supply to the DFU is often insufficient, causing cells to rely more on glycolysis for energy production, even in the presence of oxygen [52]. This process mirrors the Warburg effect, where glycolysis dominates despite available oxygen. Such a metabolic shift is not merely an adjustment but can actually hinder healing by producing energy less efficiently, increasing lactate levels, and perpetuating inflammation [53]. While such concepts are biologically plausible and supported by the preclinical literature, our study did not assess energy metabolism markers or cellular bioenergetics directly. Nevertheless, a potential reversal of the Warburg effect under HBOT is a hypothesis. HBOT may counteract this by delivering oxygen directly to the wound, potentially shifting the metabolic process back toward oxidative phosphorylation, which is more efficient for energy production [54]. This could lead to decreased lactate buildup, reduced inflammation, and improved tissue repair. As a result, the increased oxygen supply might explain the enhanced blood vessel growth and better overall healing seen in patients who received HBOT, as cells are able to function more effectively and the inflammatory environment is diminished. Moreover, by reversing the Warburg effect, HBOT could create a healthier wound environment, supporting the activity of critical repair cells like EPCs and fibroblasts [18]. This improvement in cell function and tissue health may contribute to the observed reduction in the need for major amputations among HBOT-treated patients.

Overall, these findings point to the importance of addressing the underlying metabolic imbalances in DFUs, using mechanistic studies, considering thar HBOT shows potential to correct these dysfunctions. Further research is needed to explore this connection more deeply and to develop targeted treatments aimed at restoring normal metabolic processes in chronic wounds [55].

This study presents some limitations that should be considered when interpreting the findings. The authors opted for a non-randomized trial design, which, while more feasible in real-world clinical settings, is associated with methodological limitations, namely limits causal inference and increases the potential for residual confounding. The comparison group (non-HBOT patients) was not randomly allocated but consisted of individuals who either declined HBOT or had contraindications to its use. Importantly, the refusal of HBOT was not related to clinical severity but primarily based on personal beliefs or scheduling constraints. To mitigate potential selection bias, baseline characteristics were assessed for comparability and outcome evaluations were conducted in a blinded manner.

Although the group sizes were unequal, statistical analysis confirmed comparable baseline clinical, biochemical, and wound characteristics and comorbidities. However, the small sample size and absence of a formal power calculation are also limitations. As this was an exploratory and feasibility-oriented study, participant recruitment was based on consecutive enrolment over a defined period, rather than statistical power estimation. Consequently, the study may be underpowered to detect small differences, and caution is warranted when generalizing the results. Nevertheless, the consistency of trends observed across clinical, biochemical, and histological parameters supports the relevance of the findings while highlighting the need for larger, controlled studies to confirm these results.

A further limitation concerns the reduced number of surviving or evaluable patients in the comparison group due to early major adverse outcomes (i.e., amputation or death) which may limit the robustness of long-term outcome comparisons. No multivariate analysis was performed to adjust for potential confounders, such as age, diabetes duration, or comorbidities, particularly for long-term outcomes like DFU recurrence or mortality due to the small sample size and low number of events, which would not allow for statistically reliable multivariable modelling.

Wound healing can fluctuate over time due to external or patient-related factors, which represents a potential limitation in the longitudinal interpretation of outcomes. To mitigate this, all patients received standard multidisciplinary care, and we employed consistent time points for all assessments (M0, M3, M6, M12, and M36) and ensured that the same standardized, blinded measurement techniques were used throughout. While some inter-month variability is inevitable in chronic wound healing studies, particularly over extended follow-up, the use of these mitigation measures helped reduce measurement bias.

Finally, histological analyses were limited to a small subgroup of patients, and multivariate analyses were not possible due to the limited sample size.

Taken together, these methodological considerations do not diminish the clinical interest of the findings but rather highlight the importance of validating them in larger, adequately powered, and preferably randomized studies.

## 5. Conclusions

All the presented findings reinforce the role of this treatment in diabetic patients exhibiting aggressive DFU.

Although the final sample size was limited, due to strict selection criteria, statistically significant results were observed for several outcome measures.

Patient selection for HBOT was performed by an independent team and included only patients who received optimized standard care but showed no clinical improvement. Furthermore, laboratory, molecular, and microvessels density analyses were performed by investigators blinded to the group allocation. Clinical endpoints, namely amputation and death, were objective, and all DFU measurements were performed using a digital laser measuring device.

Due to defined criteria, our diabetic foot clinic team referred for HBOT mainly DFUs that reached the bone, were infected, ischemic (grade III and stage D in the TUC), and after minor LEA has occurred. This fact may limit the results’ generalizability, and it may diminish the magnitude of effect. Accordingly, if such clinical results were observed, an even better outcome in a less severely affected population is expected.

In conclusion, our findings reinforce the potential molecular and clinical efficacy and benefit of HBOT as an adjunct to standard treatment for DFU. However, further research is needed, particularly studies involving larger cohorts in whom angiogenic and vasculogenic markers are studied not only in serum but also directly within the DFU wound bed. Additionally, investigating the modulatory effects of HBOT on DFU tissue using an animal model would help to elucidate the underlying biological pathways involved in the response to HBOT.

It is also very important to define the optimal timing of HBOT after revascularization in the treatment of DFU, as revascularization primarily improves macrovascular disease, and HBOT seems to ameliorate microvasculature. Establishing clear referral pathways in this context is equally important.

## Figures and Tables

**Table 1:** Summary of study assessments at each timepoint.

Timepoint	Baseline (M0)	1 Month (M1)	3 Months (M3)	6 Months (M6)	12 Months (M12)	36 Months (M36)
**Clinical Evaluation** **(e.g., ulcer healing, LEA, recurrence, death)**	Yes	Yes	Yes	Yes	Yes	Yes
**Digital Wound Measurement**	Yes	Yes	Yes	Yes	Yes	No
**Blood Biomarkers** **(e.g., CRP, VEGF, PlGF, SDF1-α)**	Yes	No	Yes	Yes	No	No
**Histological Analysis** ** (CD31)**	Yes (subset only)	Yes (subset only)	No	No	No	No

**Table 2:** Participants’ baseline characteristics.

Variables	Global (*n* = 20)	NHBOT DFU (*n* = 6)	HBOT DFU (*n* = 14)	*p* Value
Subject Characterization				
Age [mean (SD)]	62 (12)	63 (11)	61 (13)	0.8 *
Male gender [*n* (%)]	17 (85)	6 (100)	11 (79)	0.5 ^γ^
Visual impairment [*n* (%)]	16 (80)	5 (83)	11 (79)	1.0 ^γ^
Physical impairment [*n* (%)]	13 (65)	4 (67)	9 (64)	1.0 ^γ^
Past or present smoker [*n* (%)]	12 (60)	4 (67)	8 (57)	0.4 ^γ^
DM and Its Complications				
Type 2 [*n* (%)]	19 (95)	6 (100)	13 (93)	1.0 ^γ^
Duration (in years) [mean (SD)]	18 (9)	15 (7)	19 (10)	0.3 *
Insulin use [*n* (%)]	15 (70)	5 (83)	9 (64)	0.7 ^γ^
Cardiovascular complication history [*n* (%)]	6 (30)	1 (17)	5 (36)	0.6 ^γ^
Retinopathy complication history [*n* (%)]	18 (90)	6 (100)	12 (86)	1.0 ^γ^
Nephropathy complication history [*n* (%)]	11 (55)	5 (83)	6 (43)	0.2 ^γ^
Cerebrovascular complication history [*n* (%)]	3 (15)	0 (0)	3 (21)	0.5 ^γ^
PAD complication history [*n* (%)]	17 (85)	6 (100)	11 (79)	0.5 ^γ^
Neuropathy complication history [*n* (%)]	17 (85)	4 (67)	13 (93)	0.2 ^γ^
Metabolic complication history [*n* (%)]	9 (45)	2 (33)	7 (50)	0.6 ^γ^
Complications count [median (range)]	4 (5)	4 (2)	4 (2)	1.0 ^α^
DFU Foot Characterization				
Foot deformity [*n* (%)]	11 (55)	2 (33)	9 (64)	0.3 ^γ^
Total foot pulses ≤ 1 [*n* (%)]	19 (95)	6 (100)	13 (93)	1.0 ^γ^
ABI < 0.8 [*n* (%)]	5 (25)	2 (33)	3 (21)	0.6 ^γ^
TcPO_2_ [median (range)]	19 (67)	24 (34)	16 (67)	0.6 ^α^
Intermittent claudication [*n* (%)]	12 (60)	5 (83)	7 (50)	0.3 ^γ^
DPN symptoms [*n* (%)]	18 (90)	5 (83)	13 (93)	0.5 ^γ^
Altered SWM sensation [*n* (%)] ^a^	14 (88)	3 (60)	11 (100)	*0.08* ^γ^
Previous DFU [*n* (%)]	14 (70)	6 (100)	8 (57)	*0.1* ^γ^
Previous LEA [*n* (%)]	7 (35)	3 (50)	4 (29)	0.6 ^γ^
DFU Characterization				
Texas grade				
III (Bone or joint) [*n* (%)]	20 (100)	6 (100)	14 (100)	1.0 ^γ^
Texas stage				
B (Infection) [*n* (%)]	1 (5)	0 (0)	1 (7)	1.0 ^γ^
D (Infection plus ischemia) [*n* (%)]	19 (95)	6 (100)	13 (93)	
Located at toes [*n* (%)]	5 (25)	3 (50)	2 (14)	*0.1* ^γ^

*: Student’s *t* test for independent samples, ^γ^: Fisher’s exact test, ^α^: Mann–Whitney U test, ^a^: in 4 subjects it was not possible to conduct, ABI: Ankle-brachial index, DFU: Diabetic foot ulcer, DPN: Diabetic peripheral neuropathy, HbA_1c_: Glycated hemoglobin, HBOT: Hyperbaric oxygen therapy, LEA: Lower extremity amputation, NA: Not applicable, NHOTB: No hyperbaric oxygen therapy, PAD: Peripheral arterial disease, SD: Standard deviation, SWM: Semmes–Weistein monofilament, TcPO_2_: Transcutaneous partial pressure of oxygen.

**Table 3:** Diabetic foot ulcer characteristics at baseline and third month of follow-up.

Variables	Global (*n* = 20)		NHBOT DFU Patients (*n* = 6)		Paired Samples Tests *p* Value	HBOT DFU Patients (*n* = 14)		Paired Samples Tests *p* Value	Independent Samples Tests *p* Value	
	M0	M3	M0	M3		M0	M3		M0	M3
Baseline ulcer area (in cm^2^) [median (range)]	11.0 (31.1) ^a^	2.7 (24.8) ^b^	12.1 (16.1)	13.1 (23.5) ^b^	NP	7.3 (31.1) ^a^	2.7 (16.3)	**0.001** *	0.4 ^α^	NP
Mean depth (in mm) [median (range)]	2.3 (11.6) ^c^	1.7 (6.5) ^d^	1.8 (4.7)	3.5 (3.6) ^b^	NP	2.3 (11.6) ^c^	1.4 (6.5) ^a^	*0.1* *	0.6 ^α^	NP
Maximum depth (in mm) [median (range)]	4.6 (18.3) ^c^	3.3 (10.7) ^d^	3.6 (8.2)	7.9 (5.2) ^b^	NP	4.6 (18.1) ^c^	3.2 (10.7) ^a^	**0.03** *	0.3 ^α^	NP
Volume (in cm^3^) [median (range)]	1.2 (24.8) ^c^	0.4 (13.8) ^d^	1.5 (10.4)	7.0 (13.6) ^b^	NP	1.2 (24.5) ^c^	0.4 (10.7) ^a^	**0.006** *	0.8 ^α^	NP

*: Wilcoxon signed ranks test, ^α^: Mann–Whitney U test, ^a^: 1 missing values, ^b^: 4 missing values, ^c^: 2 missing values, ^d^: 5 missing values, cm^2^: Squared centimeter, cm^3^: Cubic centimeter, DFU: Diabetic foot ulcer, HBOT: Hyperbaric oxygen therapy, M0: Month 0, M3: Month 3, mm: Millimeter, NHBOT: No hyperbaric oxygen therapy, NP: Not possible due to patient death or lower extremity amputation.

**Table 4:** Clinical outcome.

Variables	Global (*n* = 20)			NHBOT DFU Patients (*n* = 6)			HBOT DFU Patients (*n* = 14)			Comparison Between Groups Fisher’s Exact Test *p* Value		
	M3	M6	M12	M3	M6	M12	M3	M6	M12	M3	M6	M12
Complete healing/improvement [*n* (%)]	15 (75)	15 (75)	12 (60)	1 (17)	1 (17)	1 (17)	14 (100)	14 (100)	11 (79)	**<0.001**	**<0.001**	**0.02**
Major LEA or death [*n* (%)]	5 (25)	5 (25)	8 (40)	5 (83)	5 (83)	5 (83)	0 (0)	0 (0)	3 (21)			

HBOT: Hyperbaric oxygen therapy, LEA: Lower extremity amputation, M0: Month 0, M6: Month 6, M12: Month 12, NHBOT: No hyperbaric oxygen therapy.

**Table 5:** Laboratory markers comparison intra and inter-groups.

Variables	Global			NHBOT (*n* = 6)		*p* Value	HBOT (*n* = 14)		*p* Value	NHBOT vs. HBOT *p* Value	
	M0	M3	M6	M0	M3		M0	M3		M0	M3
Glucose (mg/dL) [mean (SD)]	174 (78)	212 (82)	211 (75)	194 (110)	236 (109)	0.5 ^ε^	165 (64)	200 (68)	**0.04** ^ε^	0.5 ^ж^	0.4 ^ж^
HbA_1C_ (in %) [mean (SD)]	8.0 (1.8)	8.3 (1.8)	8.2 (1.6)	8.3 (2.1)	9.5 (2.9)	0.2 ^ε^	7.9 (1.7)	7.8 (1.1)	1.0 ^ε^	0.6 ^ж^	*0.09* ^ж^
Hemoglobin (g/dL) [mean (SD)]	12 (1)	12 (2)	12 (2)	12 (1)	11 (2)	0.3 ^ε^	12 (1)	12 (2)	0.9 ^ε^	0.8 ^ж^	0.7 ^ж^
Leukocytes (×10^3^/dL) [mean (SD)]	10.0 (3.4)	10.0 (3.7)	7.9 (1.8)	11.5 (4.1)	12.5 (4.4)	0.4 ^ε^	9.4 (3.0)	8.7 (2.7)	0.4 ^ε^	0.2 ^ж^	*0.09* ^ж^
Platelets (×10^3^/dL) [mean (SD)]	271 (74)	265 (79)	229 (76)	256 (57)	306 (90)	0.2 ^ε^	291 (76)	244 (67)	**0.04** ^ε^	0.6 ^ж^	0.2 ^ж^
Total cholesterol (mg/dL) [mean (SD)]	168 (52)	153 (37)	151 (24)	163 (68)	168 (57)	0.5 ^ε^	170 (46)	147 (26)	*0.1* ^ε^	0.8 ^ж^	0.5 ^ж^
LDL cholesterol (mg/dL) [mean (SD)]	102 (46)	84 (32)	83 (24)	97 (57)	97 (53)	0.5 ^ε^	104 (43)	79 (20)	**0.07** ^ε^	0.8 ^ж^	0.5 ^ж^
HDL cholesterol (mg/dL) [mean (SD)]	42 (9)	41 (12)	42 (17)	43 (10)	36 (13)	0.1 ^ε^	41 (9)	43 (11)	0.6 ^ε^	0.7 ^ж^	0.3 ^ж^
Triglycerides (mg/dL) [mean (SD)]	131 (56)	143 (85)	131 (70)	112 (44)	177 (82)	0.1 ^ε^	133 (60)	130 (86)	0.9 ^ε^	0.5 ^ж^	0.3 ^ж^
Uric acid (mg/dL) [mean (SD)]	5 (2)	6 (2)	6 (2)	5 (5)	7 (7)	**0.04** ^ε^	5 (6)	5 (6)	0.3 ^ε^	0.9 ^ж^	0.2 ^ж^
Urea (mg/dL) [mean (SD)]	58 (25)	69 (47)	63 (34)	66 (33)	92 (65)	0.1 ^ε^	54 (21)	57 (32)	0.5 ^ε^	0.4 ^ж^	0.1^ж^
Creatinine (mg/dL) [median (range)]	1.4 (7.3)	1.5 (8.2)	1.1 (0.6)	1.3 (7.3)	1.6 (8.2)	0.2 ^Ϫ^	1.0 (1.8)	0.9 (2.9)	0.6 ^Ϫ^	0.6 ^α^	1.0 ^α^
Microalbuminuria [*n* (%)]	11 (55)	12 (60)	9 (45)	4 (67)	4 (67)	1.0 ^γ^	7 (50)	8 (57)	0.9 ^γ^	0.3 ^γ^	0.6 ^γ^
Erythrocyte sedimentation rate (mm) [median (range)]	65 (97)	37 (111)	42 (107)	104 (32)	33 (16)	**0.02**	58 (72)	30 (111)	**0.04** **^Ϫ^**	0.1 ^α^	0.6 ^α^
C-reactive protein (mg/dL) [median (range)]	0.9 (18)	0.3 (51)	0.3 (9)	2.1 (11)	11.8 (22.4)	0.1 ^Ϫ^	0.7 (15.7)	0.3 (5.4)	*0.08* ^Ϫ^	0.2 ^α^	**0.03** ^α^
VEGF (pg/mL) [median (range)]	90 (236)	63 (344)	47 (29)	189 (236)	99 (344)	0.9 ^Ϫ^	72 (268)	56 (129)	*0.06* ^Ϫ^	0.6 ^α^	*0.06* ^α^
PlGF (pg/mL) [median (range)]	13.4 (66.0)	8.5 (65.8)	10.5 (51.2)	3.1 (14.7)	7.4 (12.1)	0.7 ^Ϫ^	14.8 (65.7)	9.6 (64.0)	*0.08* ^Ϫ^	0.2 ^α^	0.4 ^α^
SDF1-α (pg/mL) [mean (SD)]	1890 (687)	2132 (563)	1738 (709)	2297 (514)	2455 (476)	0.4 ^ε^	1716 (691)	2017 (562)	*0.1* ^ε^	*0.08* ^ж^	0.1 ^ж^

^ε^: Student’s *t* test for paired samples, ^γ^: X^2^ test, ^Ϫ^: Wilcoxon signed ranks test, ^ж^: Student’s *t* test for independent samples, ^α^: Mann–Whitney U test, %: Percentage, DFU: Diabetic foot ulcer, dl: Deciliter, g: Gram, HbA_1c_: Glycated hemoglobin, HBOT: Hyperbaric oxygen therapy, HDL: High-density lipoprotein, LDL: Low-density lipoprotein, M0: Month 0, M3: Month 3, M6: Month 6, mg: Milligram, ml: Milliliter, NA: Not applicable, NHOTB: No hyperbaric oxygen therapy, pg: Picogram; PlGF: Placental growth factor, SD: Standard deviation, SDF1-α: Stromal cell-derived factor 1 alpha, VEGF: Vascular endothelial growth factor.

## Data Availability

Data are unavailable due to privacy restrictions.

## Abbreviations

The following abbreviations are used in this manuscript:

ABC	Avidin Biotin Complex
ABI	Ankle Brachial Index
ATA	Atmospheres Absolute
BSI	Bovine Serum Albumin
CD	Cluster of Differentiation
CRP	C-reactive Protein
DAB	Diaminobenzidine
DFU	Diabetic Foot Ulcer
DM	Diabetes Mellitus
DPN	Diabetic Peripheral Neuropathy
EDTA	Ethylenediamine Tetraacetic Acid
ELISA	Enzyme-linked Immunosorbent Assay
EPC	Endothelial Progenitor Cells
ESR	Erythrocyte Sedimentation Rate
HbA1C	Glycated Hemoglobin A
HBOT	Hyperbaric Oxygen Therapy
HDL	High-density lipoprotein
H&E	Hematoxylin and Eosin
kPa	Kilopascal
LDL	Low-density Lipoprotein
LEA	Lower Extremity Amputation
mg/dL	Milligrams per deciliter
NHBOT	Non- Hyperbaric Oxygen Therapy
NRT	Non-randomized Trial
PlGF	Placental Growth Factor
PAD	Peripheral Arterial Disease
PBS	Phosphate Buffered Saline
pO2	Tissue Oxygen Pressure
RCT	Randomized Controlled Trial
SD	Standard Deviation
SDF1-α	Stromal-derived Factor-Alpha
SWM	Semmes–Weinstein Monofilament
TcPO2	Transcutaneous Partial Pressure of Oxygen
TUC	Texas University Classification
UK	United Kingdom
VEGF	Vascular Endothelial Growth Factor
